# Native Collagen for Surgical Wound and Scar Prevention—A Six-Case Clinical Series

**DOI:** 10.3390/jcm14196989

**Published:** 2025-10-02

**Authors:** Olga B. Borzykh, Elena I. Karpova, Marina M. Petrova, Natalia A. Shnayder, Svetlana V. Danilova

**Affiliations:** 1 Plastic Surgery and Cosmetology Clinic “Dr. Albrecht”, Voronezh 394036, Russia; 2 Department of Plastic, Reconstructive Surgery, Cosmetology and Cellular Technologies, Pirogov Russian National Research Medical University, Moscow 119435, Russia; elena-karpova@inbox.ru; 3 Shared Core Facilities “Molecular and Cell Technologies”, Voino-Yasenetsky Krasnoyarsk State Medical University, Krasnoyarsk 660022, Russia; stk99@yandex.ru (M.M.P.); naschnaider@yandex.ru (N.A.S.); 4 Institute of Personalized Psychiatry and Neurology, Shared Core Facilities, V. M. Bekhterev National Medical Research Centre for Psychiatry and Neurology, Saint Petersburg 192019, Russia; 5 Galaktika Clinic, Moscow 121205, Russia; danilovamezo@mail.ru

**Keywords:** collagen, wound healing, scar, plastic surgery, hypertrophic scar

## Abstract

**Background:** Excessive scarring remains a frequent complication in plastic surgery, yet standardized preventive strategies are lacking. Type I collagen-based biomaterials may support regenerative processes and improve scar outcomes. **Methods:** This case series includes six female patients (ages 24–52) undergoing wound management after trauma and procedures including blepharoplasty, abdominoplasty, and revision mammaplasty. Native collagen type I (7% or 15%) was injected along wound margins or into hypertrophic scars at 3–4 week intervals. Outcomes were assessed through patient-reported symptoms and Antera 3D imaging (vascularity, pigmentation, surface topography). **Results:** Patients reported reduced tightness, pruritus, and scar stiffness after initial sessions. Antera 3D imaging showed decreased vascular and pigment indices, and a reduction in surface elevation over follow-up (up to 14 months). No adverse effects such as atrophy or infection were observed. **Conclusions:** Native type I collagen was well tolerated and may be a useful adjunct for wound healing and scar modulation following plastic surgery.

## 1. Introduction

Despite numerous studies focused on hypertrophic scars and the possibilities of scarless wound healing, excessive postoperative or post-traumatic scarring remains a challenge [1]. The issue is also highly relevant due to its prevalence [2,3]. Hypertrophic scar formation usually begins 1–2 months after injury, enters a phase of rapid growth (lasting 6–18 months), and may take several years to regress [4]. For physicians across specialties, it is crucial not only to control infection and close the wound but also to actively manage the wound healing process [5]. Wound healing is a complex and finely regulated biological process involving the transition from clot formation to granulation tissue. This process requires a balance between the deposition and degradation of extracellular matrix (ECM) proteins. Disruption of this balance can result in abnormal scar formation [1].

Various invasive (e.g., platelet-rich plasma injections, botulinum toxin type A, corticosteroids, 5-fluorouracil, interferons, and bleomycin) [6] and non-invasive (e.g., laser therapy, topical growth factor and cytokine formulations, silicone-based products) [4,7] approaches have been proposed to improve wound healing and control scarring. However, no universal gold standard has yet been established [8]. The general concept behind wound healing strategies is to accelerate healing and prevent excessive scarring. Paradoxically, the same growth factors that promote healing can furthermore increase the risk of scarring. Furthermore, several treatments are associated with adverse effects that may negatively impact wound healing [9,10].

The most widely used treatments for hypertrophic scars include intralesional corticosteroids, botulinum toxin type A, and laser therapy. However, none of these methods are capable of fully preventing excessive scar formation or regenerating healthy dermal tissue. Once scar tissue matures, the regeneration of normal dermis from surrounding tissue becomes impossible [4]. Additionally, the total wound healing time is one of the most critical factors in predicting the development of hypertrophic scars [4]. Therefore, new approaches aim to enhance tissue regeneration and accelerate healing.

Some authors attribute the primary cause of hypertrophic scarring to intense inflammation. As preventive measures, they recommend silicone-based and anti-inflammatory dressings, while corticosteroid injections are considered the mainstay treatment [1,4,6,11,12,13,14,15,16,17,18].

Limitations of corticosteroid use include potential side effects and their lack of impact on regeneration or healing speed. Adverse effects may include skin atrophy, telangiectasia, injection site pain, and systemic effects like osteoporosis. Higher doses tend to increase efficacy however furthermore also raise the risk of side effects. Thus, corticosteroids are considered a second-line treatment by numerous authors (when other methods are ineffective) [1,19].

Due to the absence of a gold standard for scar management, researchers are actively exploring novel agents that offer improved efficacy and safety [20]. These agents target different stages of wound healing—from hemostasis to ECM remodeling—by promoting angiogenesis, immune modulation, and granulation tissue formation. However, despite the development of new products, significant problems remain in wound treatment [21]. Despite recent advances, there is still no treatment method that would completely prevent the formation of scars and stimulate the regeneration of skin appendages [22].

Biopolymer-based wound dressings, such as those made from dextran and chitosan, have demonstrated efficacy in promoting wound healing, angiogenesis, and regulating collagen synthesis. It furthermore improves the structure of the formed collagen, reducing its volume around the damaged area. These scaffolds mimic the natural ECM, providing mechanical support, cellular adhesion, hydration, and structural guidance for tissue regeneration [22,23,24]. Key components of the dermal microstructure, such as collagen, elastin, hyaluronic acid, fibronectin, perlecan, and water, exist in a specific three-dimensional organization that supports interactions between epidermal and dermal cells. This interaction affects attachment, migration, differentiation, and morphogenetic phenomena. Thus, the EMC promotes “proper” connections between keratinocytes and fibroblasts and is furthermore responsible for the formation and maintenance of skin appendages. Disruption of this matrix impairs skin appendage formation and epidermal self-renewal. Dysfunctional ECM remodeling contributes to pathological scarring [24].

Skin substitutes incorporating cells, cytokines, and ECM-mimicking materials—especially those based on collagen—have been used to stimulate regeneration. The most commonly used matrix is based on collagen (it is completely biocompatible and decomposable). These scaffolds form the basis for revascularization, forming an appropriate microenvironment for cell migration and proliferation. The same property of collagen is the basis for its use in the manufacture of scaffolds (collagen hydrogels, collagen scaffolds made of microfiber, nanofiber scaffolds made of electrically spun collagen) used to improve wound regeneration [25].

Collagen type I acts as a biological modulator of the wound environment, promoting epithelialization, fibroblast activity, and angiogenesis [25]. In animal models, collagen has reduced inflammation and improved wound closure [26]. Re-epithelialization is the most important stage involving the migration and proliferation of keratinocytes and skin fibers. The cultivation of cells with collagen demonstrated an increase in the proliferation rate. Thus, in the early stages of wound healing, collagen promotes the intensive proliferation of immortalized epidermal cells and fibroblasts. With further differentiation of fibroblasts into myofibroblasts, contributing to the narrowing of the wound. Collagen has furthermore been shown to stimulate cell migration and accelerate healing. The cultivation of cell cultures with collagen significantly increased the expression of Col α(I) and TGF-β1. At a later stage, the expression of Col α(I) and TGF-β1 proteins was furthermore significantly increased. There was also an increase in VIM (vimentin), which supports the stability of the cytoskeleton, indicating the role of collagen in ensuring cell stability. Histological examination demonstrated that on day 8, fewer inflammatory cells were found in the collagen group, and by day 13, greater angiogenesis (compared with the saline group). Collagen enhances *VEGF* gene expression and affects various wound healing processes such as angiogenesis, re-epithelialization, and collagen synthesis [26].

Using a model of an ischemic wound in rats, it was shown that the use of a product based on native collagen of 7% promotes the accelerated appearance of M2 macrophages with anti-inflammatory and pro-regenerative activity. Also, in wounds using a product based on native collagen of 7%, there was a decrease in the intensity and duration of inflammation, acceleration of angiogenesis, and a change in the expression of growth factors (a faster increase immediately after injury, followed by a decrease). Such a change in the expression of growth factors led to a faster and more efficient process of re-epithelialization, neovasculogenesis, and closure of the wound defect, without the development of prolonged inflammation and wound proliferation [27].

The use of native collagen to control wound healing is a promising area; however, there is currently a lack of clinical studies on efficacy and safety. In this article, we present our clinical experience of using a product based on native collagen in the practice of the Plastic surgery and cosmetology clinic. We selected a product based on native bovine collagen (Collost gel, BioPHARMAHOLDING, Moscow, Russia) as the main treatment. The product is a hydrogel based on collagen fibrils, with a concentration of collagen of 7 and 15%, dissolved in a glucose solution of 5%. The collagen gel was administered intradermally and intralesionally. The choice of collagen concentration and volume of product depended on the area of the scar and the intensity of scarring. To assess the dynamics of treatment, we used photographs taken under the same conditions and 3D photographs taken using the Antera 3D Camera (Miravex, Dublin, Ireland). The latter allow for a more objective assessment of changes in the color, volume, and vascular component of the scar. When using Antera 3D, the Automatic Matching mode was used. Which automatically matches two or more images to each other. Automatic matching automatically compensates for relative shifts and rotations of images, which allows you to obtain reliable results. In addition, the change in subjective symptoms and the assessment of scars on the Vancouver scar scale were evaluated [28].

## 2. Clinical Cases

### 2.1. Case Presentation 1: Wound Healing

**Figure 1:**
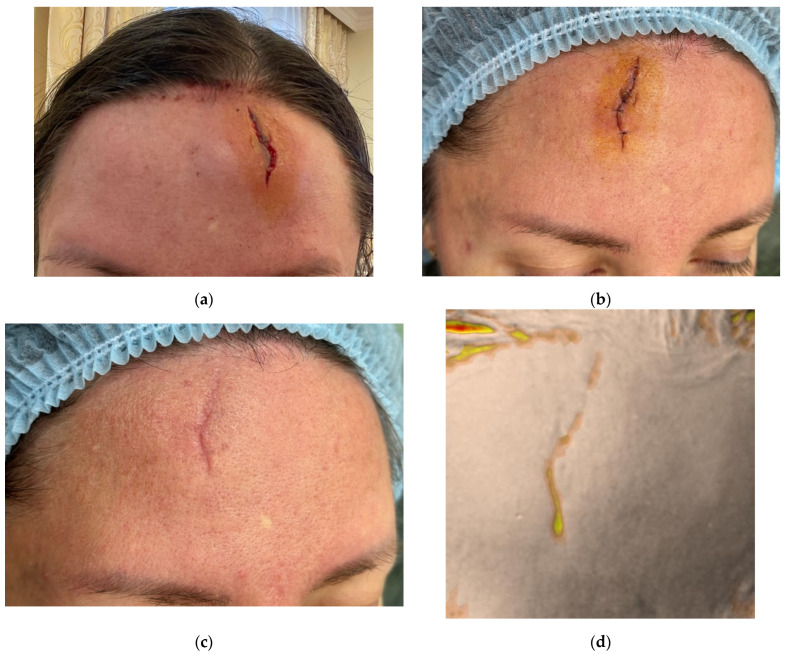

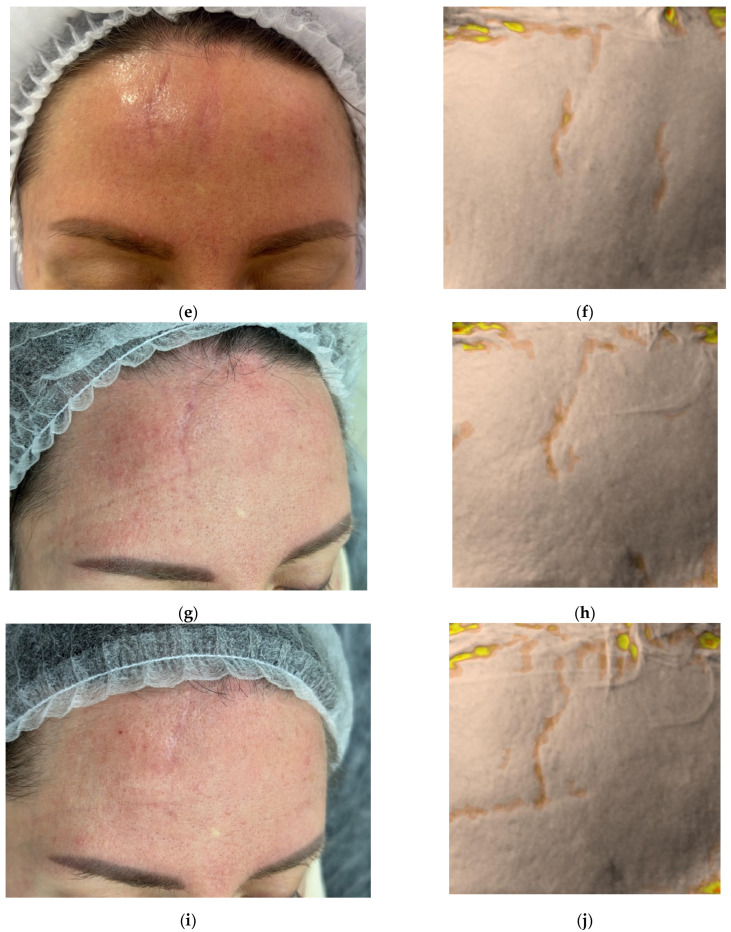

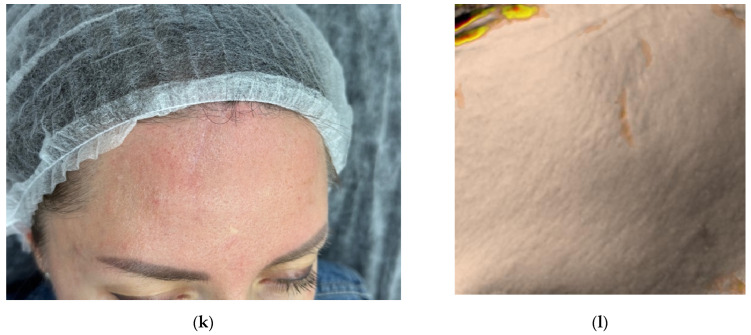
Clinical photographs (**a**–**c**,**e**,**g**,**i**,**k**) and Antera 3D surface topography maps (**d**,**f**,**h**,**j**,**l**) illustrating the progressive healing of a traumatic facial wound in a 41-year-old female. Images show baseline (Day 0, post-injury) (**a**), Day 2 (pre-treatment) (**b**), Day 17 (proliferation phase) (**c**,**d**), Day 34 (early remodeling) (**e**,**f**), 2.5 months (**g**,**h**), 3.5 months (**i**,**j**), and 4.5 months post-injury (**k**,**l**). Antera 3D maps visualize scar volume.

A 41-year-old female patient presented with the goal of improving wound healing. Two days earlier, she had sustained a laceration to the forehead approximately 30 mm in length (Figure 1a). Six hours after the injury, the wound was cleaned and closed with simple interrupted sutures. Given the positive experimental results with native collagen in early wound healing, a 0.5 mL dose of 7% native collagen was administered on the day of presentation.

On Day 2, during the inflammatory phase of healing, the wound demonstrated swelling at the edges however no granulation tissue (Figure 1b). After antiseptic treatment, the collagen gel was injected intradermally around the wound margins, with the remaining gel distributed within the wound gap. A sterile dressing was applied. Sutures were removed on Day 10.

The second application of collagen was performed on Day 17 during the proliferative phase (Figure 1c,d). A 0.5 mL dose of 7% collagen was injected intradermally along the wound margins and directly into the granulating tissue (intralesional).

The third procedure was performed on Day 34, during the remodeling phase (Figure 1e,f). The collagen (0.5 mL, 7%) was again administered intradermally and intralesional. Two additional procedures were performed at 2.5 and 3.5 months post-injury. On follow-up at 4.5 months, the patient presented with a thin, pale, normotrophic scar (The Vancouver scar score scale is 1).

**Figure 2:**
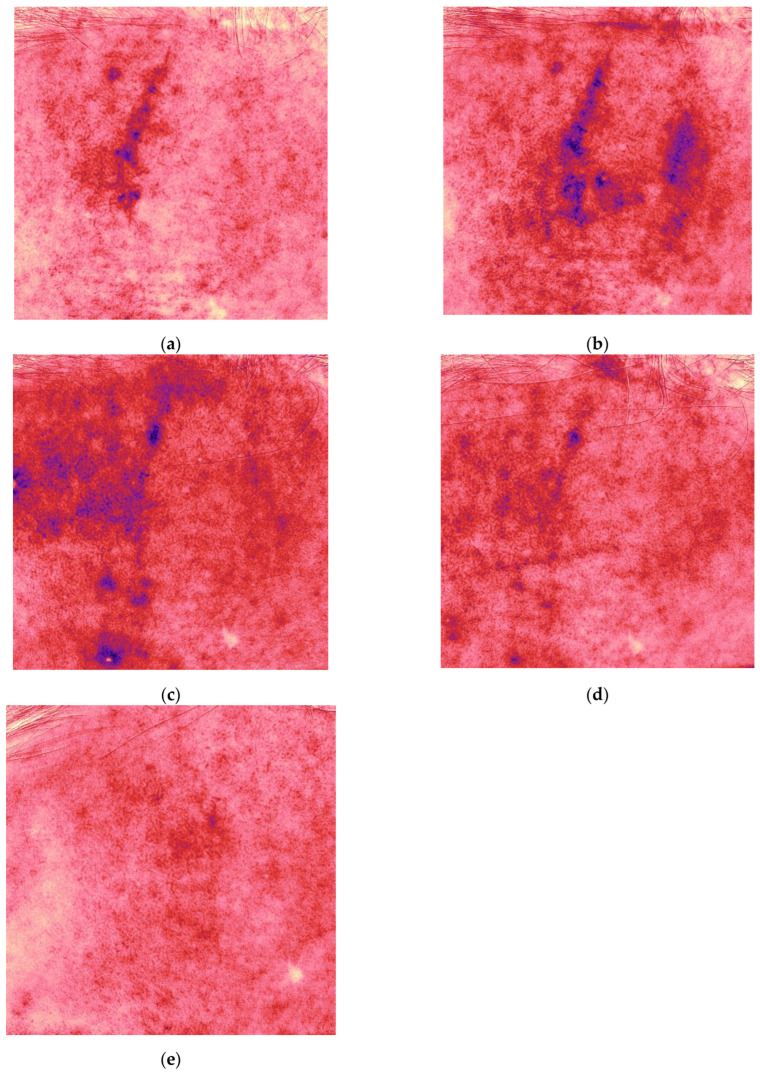
Redness (vascularity) maps acquired with Antera 3D for the same patient and time points shown in Figure 1 ((**a**) Day 17, (**b**) Day 34, (**c**) 2.5, (**d**) 3.5, and (**e**) 4.5 months).

Antera 3D imaging demonstrated an increase in the depth, width and redness on Day 34, followed by a significant decrease at 4.5 months (Figure 2, Table 1).

Throughout the treatment, the patient did not experience such subjective sensations as itching, tightening, etc. The patient remains under observation.

### 2.2. Case Presentation 2: Wound Healing After Upper Eyelid Blepharoplasty

**Figure 3:**
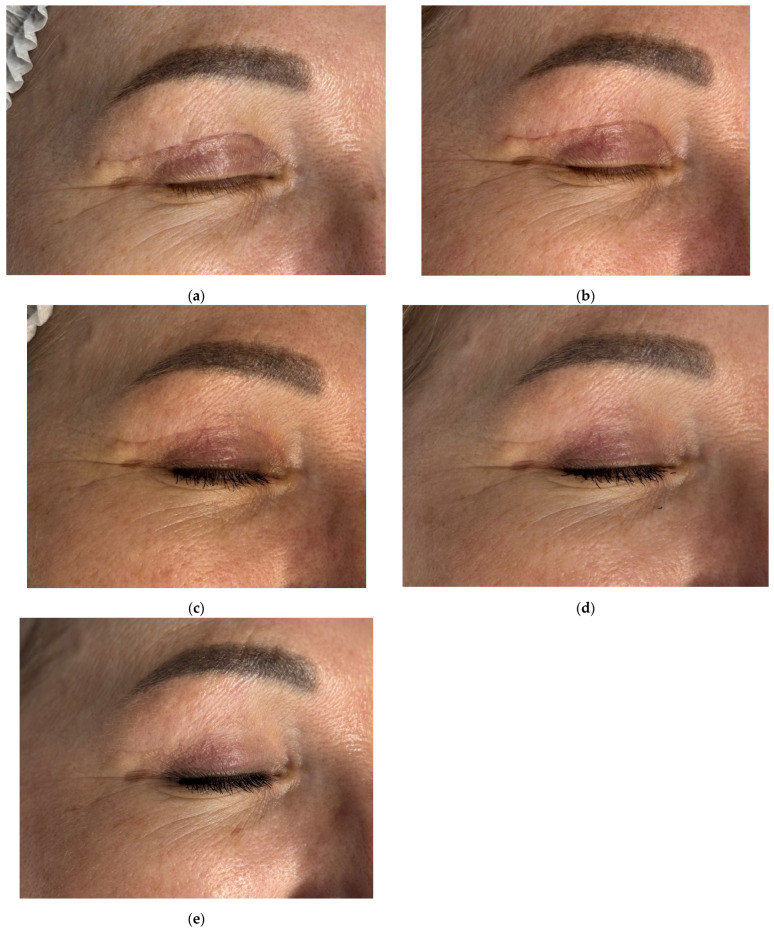
Clinical follow-up photographs of upper eyelid postoperative scar in a 56-year-old female patient after blepharoplasty. Images correspond to (**a**) 4 weeks after upper eyelid blepharoplasty (baseline, pre-treatment); (**b**) 6 weeks after upper eyelid blepharoplasty (before treatment); (**c**) 9 weeks after upper eyelid blepharoplasty (3 weeks after procedure); (**d**) 12 weeks after upper eyelid blepharoplasty (3 weeks after second procedure); (**e**) 16 weeks after upper eyelid blepharoplasty (the result after 3 procedures).

A 56-year-old female patient presented for follow-up 4 weeks after upper eyelid blepharoplasty (Figure 3a). Standard postoperative care included topical corticosteroids (2 weeks), followed by enzymatic agents (2 weeks), then silicone-based and SPF 50 sunscreen creams.

**Figure 4:**
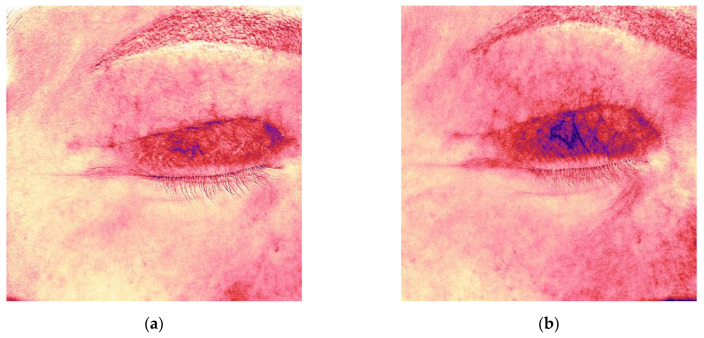

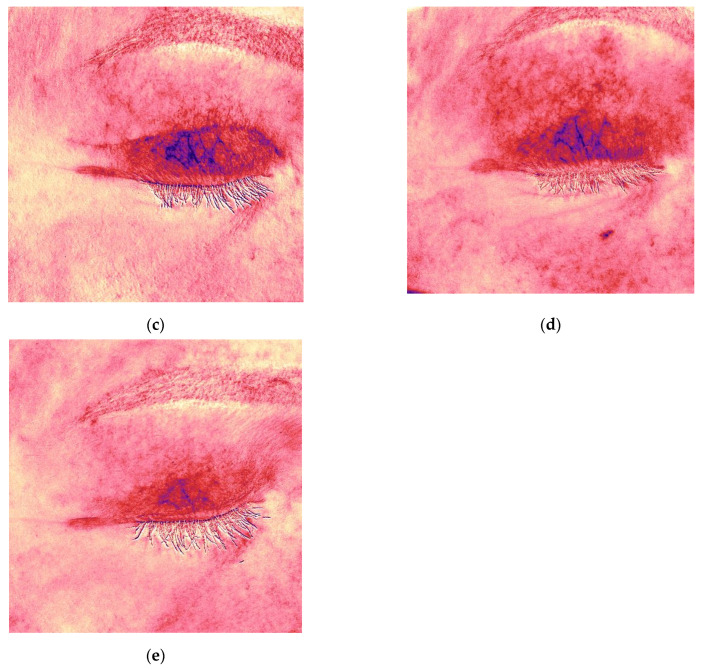
Antera 3D redness maps for the upper eyelid scar of the same patient, demonstrating progressive decrease in vascularity at 4, 6, 9, 12, and 16 weeks post-surgery. (**a**) 4 weeks after upper eyelid blepharoplasty; (**b**) 6 weeks after upper eyelid blepharoplasty (before treatment); (**c**) 9 weeks after upper eyelid blepharoplasty (3 weeks after procedure); (**d**) 12 weeks after upper eyelid blepharoplasty (3 weeks after second procedure); (**e**) 16 weeks after upper eyelid blepharoplasty (the result after 3 procedures).

Initial Antera 3D imaging revealed a moderate vascular component in the scar (Figure 4a). At the 6-week visit, the patient reported pruritus and slight thickening of the scar, which furthermore appeared redder (Figure 3b). Antera 3D confirmed increased vascularity (Figure 4b, Table 2). The Vancouver scar score scale is 4.

To improve healing and scar modulation, 0.5 mL of 7% native collagen was injected intradermally, intralesional, and into the periorbital skin for rejuvenation. A total of three procedures were performed at 3-week intervals.

Following the first procedure, the patient noted resolution of pruritus. By the second visit, redness and vascularity had decreased (Figure 3c and Figure 4c). After three treatments, the scars appeared thin, normotrophic, and nearly invisible (Figure 3e). Skin tone in the upper eyelid area was even, and periorbital wrinkles were reduced. The Vancouver scar score scale is 0.

Antera 3D imaging confirmed a steady reduction in vascularity over time (Figure 4c–e, Table 2).

The patient remains under observation.

### 2.3. Case Presentation 3: Treatment of a Hypertrophic Scar Following Upper Eyelid Blepharoplasty

**Figure 5:**
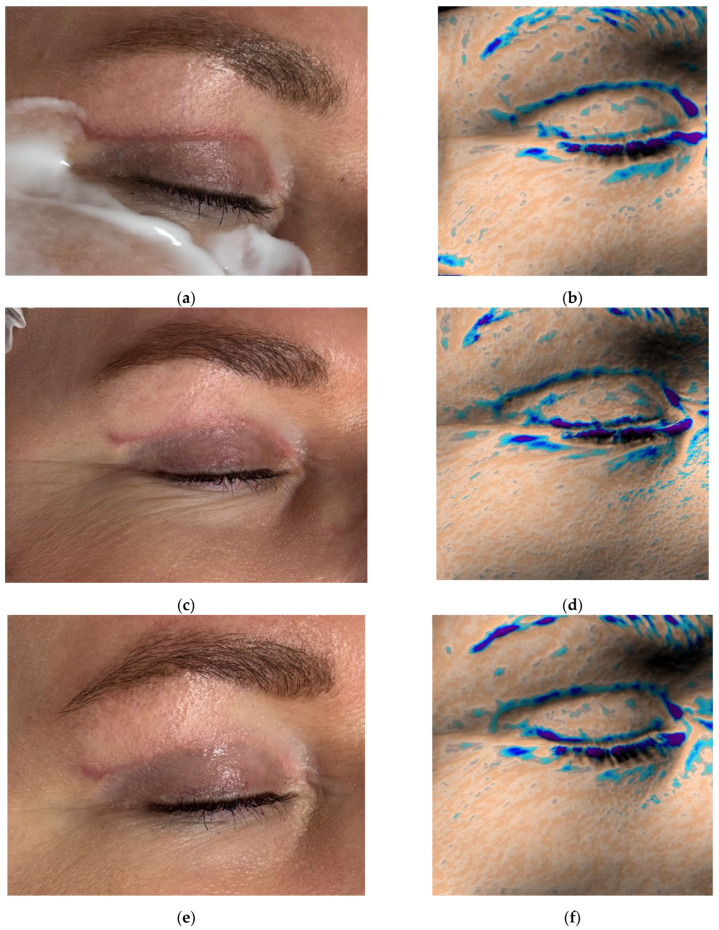

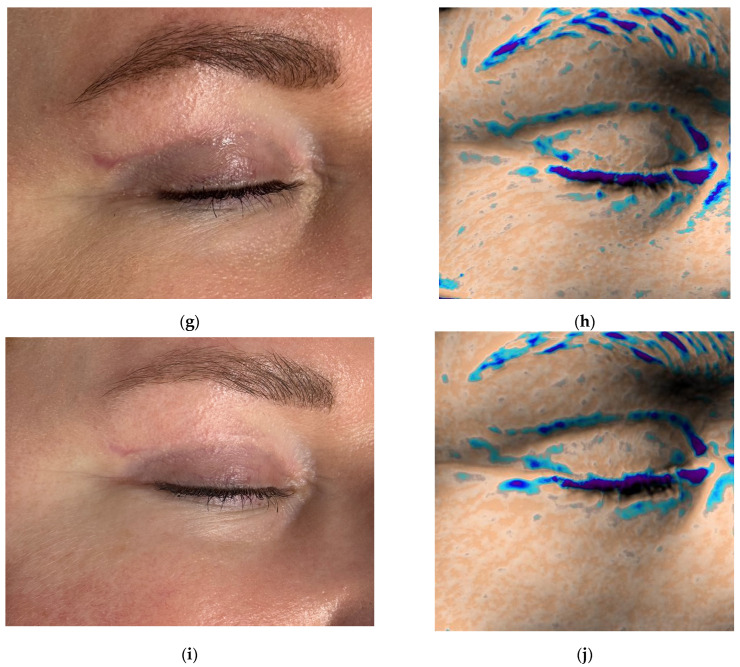
Clinical photographs (**a**,**c**,**e**,**g**,**i**) and Antera 3D topography maps (**b**,**d**,**f**,**h**,**j**) showing hypertrophic scar evolution in a 50-year-old female after blepharoplasty. Imaging demonstrates (**a**,**b**) 5 weeks after upper eyelid blepharoplasty (baseline); (**c**,**d**) 8 weeks after upper eyelid blepharoplasty (3 weeks after procedure); (**e**,**f**) 11 weeks after upper eyelid blepharoplasty (3 weeks after second procedure); (**g**,**h**) 15 weeks after upper eyelid blepharoplasty (3 weeks after third procedure); (**i**,**j**) 19 weeks after upper eyelid blepharoplasty (the result after 4 procedures).

A 50-year-old female patient presented with complaints of rapid scar growth, pruritus, and a pulling sensation in the upper eyelid area. Five weeks earlier, she had undergone upper eyelid blepharoplasty (Figure 5a). The Vancouver scar score scale is 7. Standard postoperative care included topical corticosteroids (2 weeks), enzyme-based creams (2 weeks); and silicone cream with SPF 50 sunscreen cream after four weeks.

**Figure 6:**
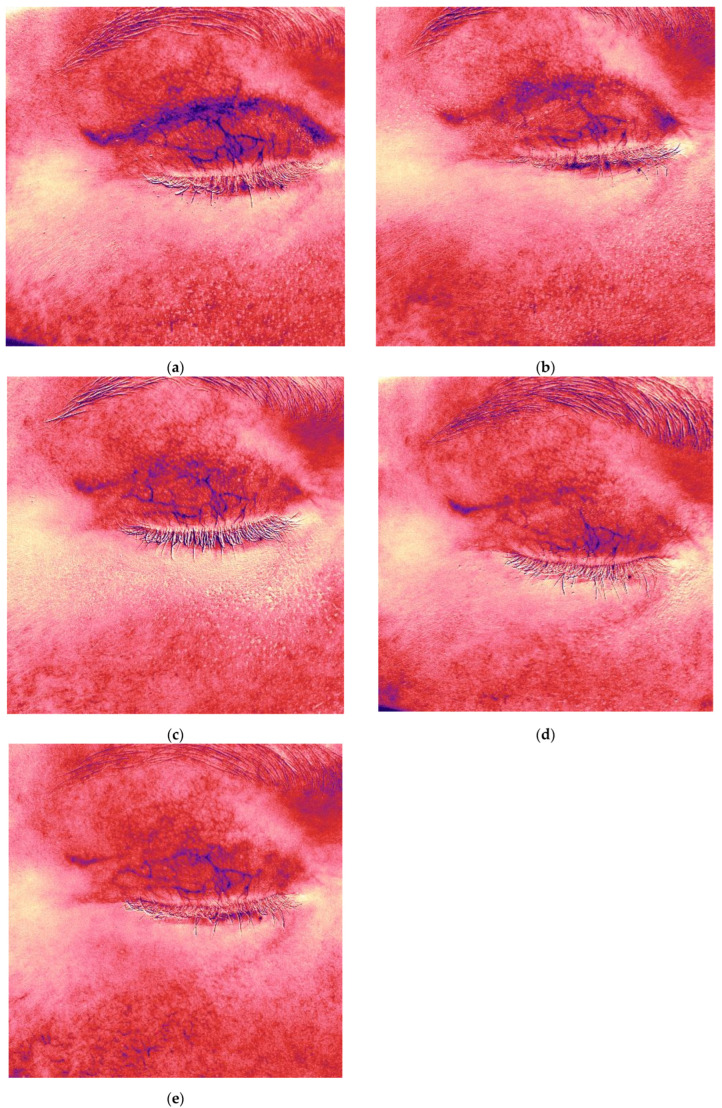
Antera 3D redness maps of the hypertrophic eyelid scar from Figure 5, showing stepwise reduction in vascularity at each follow-up (5, 8, 11, 15, 19 weeks post-surgery). Imaging demonstrates (**a**,**b**) 5 weeks after upper eyelid blepharoplasty (baseline); (**c**,**d**) 8 weeks after upper eyelid blepharoplasty (3 weeks after procedure); (**e**) 11 weeks after upper eyelid blepharoplasty (3 weeks after second procedure).

Antera 3D imaging revealed tissue overgrowth at the scar site and increased vascularity (Figure 5b and Figure 6a). A diagnosis of hypertrophic scarring was made.

The patient received 0.5 mL of 7% native collagen injected intradermally, intralesional, and periorbitally for skin rejuvenation. Four procedures were performed at 3-week intervals.

Following the first treatment, the patient reported cessation of itching. By the second visit, redness and vascularity had decreased (Figure 5c and Figure 6b). After four treatments, the scars became flatter and less noticeable (Figure 5i). The Vancouver scar score scale is 2. Progressive reduction in scar volume and vascular intensity (redness) was confirmed on Antera 3D (Figure 5b,d,f,h,j and Figure 6a–e, Table 3).

The patient remains under observation.

### 2.4. Case Presentation 4: Treatment of a Hypertrophic Scar and Postoperative Hyperpigmentation Following Upper Eyelid Blepharoplasty

**Figure 7:**
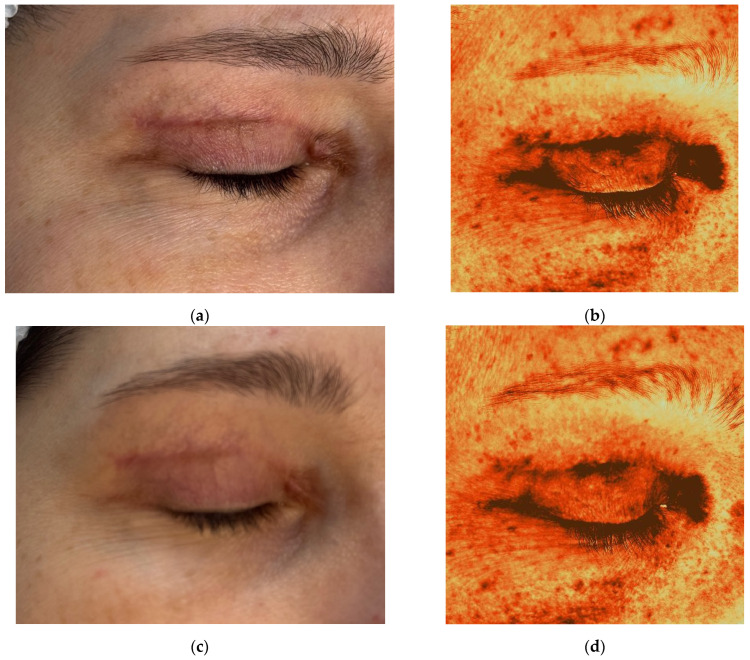

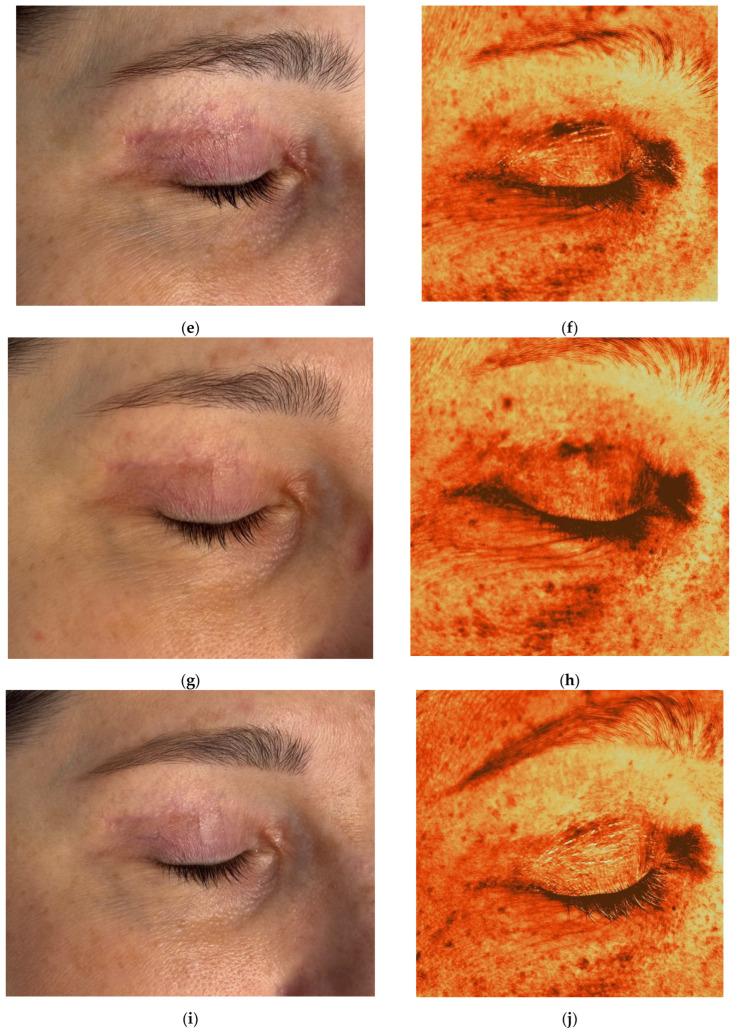
Clinical photographs (**a**,**c**,**e**,**g**,**i**) and Antera 3D melanin maps (**b**,**d**,**f**,**h**,**j**) illustrating treatment response in a 45-year-old female with postoperative hyperpigmented hypertrophic scar after blepharoplasty. Time points: (**a**,**b**) 6 weeks after upper eyelid blepharoplasty; (**c**,**d**) 9 weeks after upper eyelid blepharoplasty (3 weeks after procedure); (**e**,**f**) 12 weeks after upper eyelid blepharoplasty (3 weeks after second procedure); (**g**,**h**) 16 weeks after upper eyelid blepharoplasty (3 weeks after third procedure); (**i**,**j**) 20 weeks after upper eyelid blepharoplasty (the result after 3 procedures).

A 45-year-old female patient presented with complaints of rapid scar growth over the past week, darkening of the skin in the scar area over the past two weeks, itching, and a sensation of tightness. She had undergone upper eyelid blepharoplasty six weeks earlier (Figure 7a). Standard postoperative care included topical corticosteroids (2 weeks), enzyme cream (2 weeks), and later silicone-based cream and SPF 50 sunscreen.

**Figure 8:**
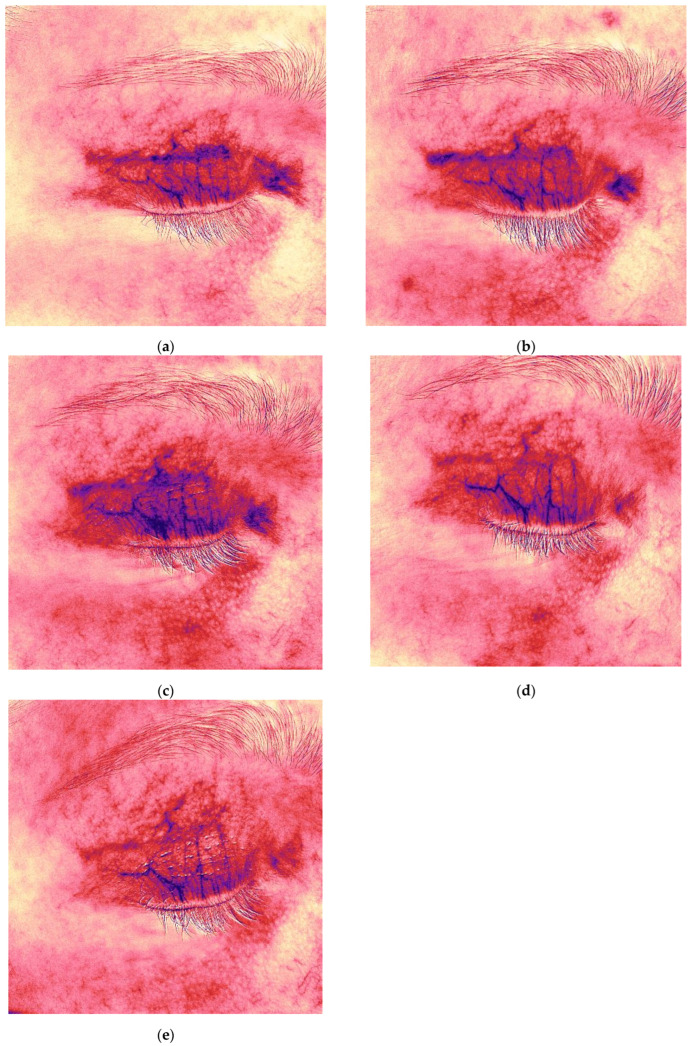
Redness (vascularity) maps of the hyperpigmented hypertrophic scar from Figure 7, demonstrating decreased vascularity after collagen therapy (6 → 20 weeks post-surgery). Time points: (**a**,**b**) 6 weeks after upper eyelid blepharoplasty; (**c**,**d**) 9 weeks after upper eyelid blepharoplasty (3 weeks after procedure); (**e**) 12 weeks after upper eyelid blepharoplasty (3 weeks after second procedure).

On examination, there was significant hyperpigmentation in the scar region and at the outer corner of the eye. The patient reported a postoperative hematoma in the lateral canthal area. Thickening of the scar was observed medially. The Vancouver scar score scale is 8. Antera 3D imaging revealed severe pigmentation, especially in the medial scar and outer eye area (Figure 7b), and severe redness (vascularity) (Figure 8a, Table 4). A diagnosis of postoperative hyperpigmentation and hypertrophic scarring was made.

To improve wound healing and manage scarring, 0.5 mL of 7% native collagen was injected intradermally, intralesional, and periorbitally for skin rejuvenation. The patient was furthermore advised to wear sunglasses for sun protection. Three treatments were performed at three-week intervals.

After the first treatment, the patient reported relief from itching and tightness. At the second visit, decreased pigmentation and softening of the medial scar were noted (Figure 7c). Two months after completing treatment, the scars appeared thin and normotrophic, pigmentation was reduced, and periorbital skin quality improved, including decreased wrinkles and improved tone (Figure 7i). The Vancouver scar score scale is 3. Antera 3D imaging demonstrated a gradual reduction in pigmentation and vascularity, with the latter beginning to decrease after the third procedure (Figure 7b,d,f,h,j and Figure 8a–e, Table 4).

The patient remains under observation. Azelaic acid cream was added to the home care regimen for additional depigmenting and anti-inflammatory effects.

### 2.5. Case Presentation 5: Treatment of a Hypertrophic Scar After Abdominoplasty

**Figure 9:**
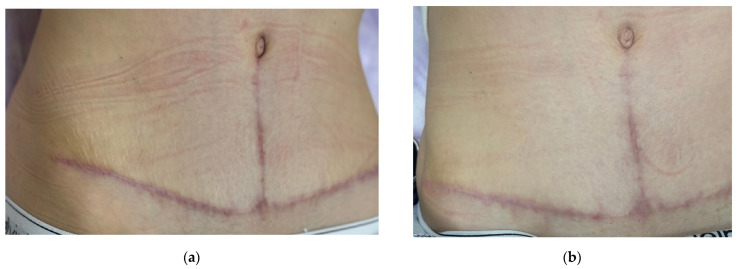

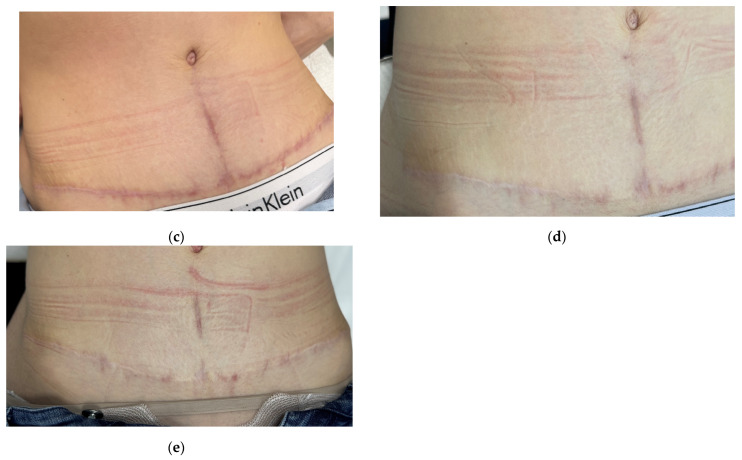
Sequential clinical photographs of a hypertrophic abdominal scar in a 34-year-old female patient. Photos show (**a**) baseline at 3 months after surgery (before treatment); (**b**) 4 months after surgery (3 weeks after the first procedure); (**c**) 4.5 months after surgery (3 weeks after the second procedure); (**d**) 6 months after surgery (1.5 months after the third procedure); (**e**) 9 months after surgery (3 months after the fourth procedure).

A 34-year-old female patient complained of scar enlargement over the past month, itching, and tension. She had undergone abdominoplasty three months earlier (Figure 9a). Standard postoperative care included topical corticosteroids (2 weeks), followed by enzyme creams (2 weeks) and later silicone-based products.

Examination revealed a dense, vertically prominent scar with overall postoperative erythema. The Vancouver scar score scale is 7. A diagnosis of postoperative hypertrophic scarring was made.

Initially, 1 mL of 15% native collagen was injected intralesional throughout the scar tissue. The patient reported reduced itching and tightness after the first session. By three weeks, there was noticeable fading of erythema and decreased density of the vertical scar (Figure 9b).

Two more treatments followed at three-week intervals using 1.5 mL of 7% collagen. At the three-month follow-up, erythema had further decreased, although a 4 cm vertical hypertrophic scar remained (Figure 9d). A fourth procedure was performed with 1.5 mL of 7% collagen.

After 3 months, a decrease in the intensity of vertical hypertrophic scar was noted. The rest of the scar after abdominoplasty is white and barely noticeable. The Vancouver scar score scale is 4.

The patient remains under observation.

### 2.6. Case Presentation 6: Prevention of Hypertrophic Scarring After Revision Mammaplasty

**Figure 10:**
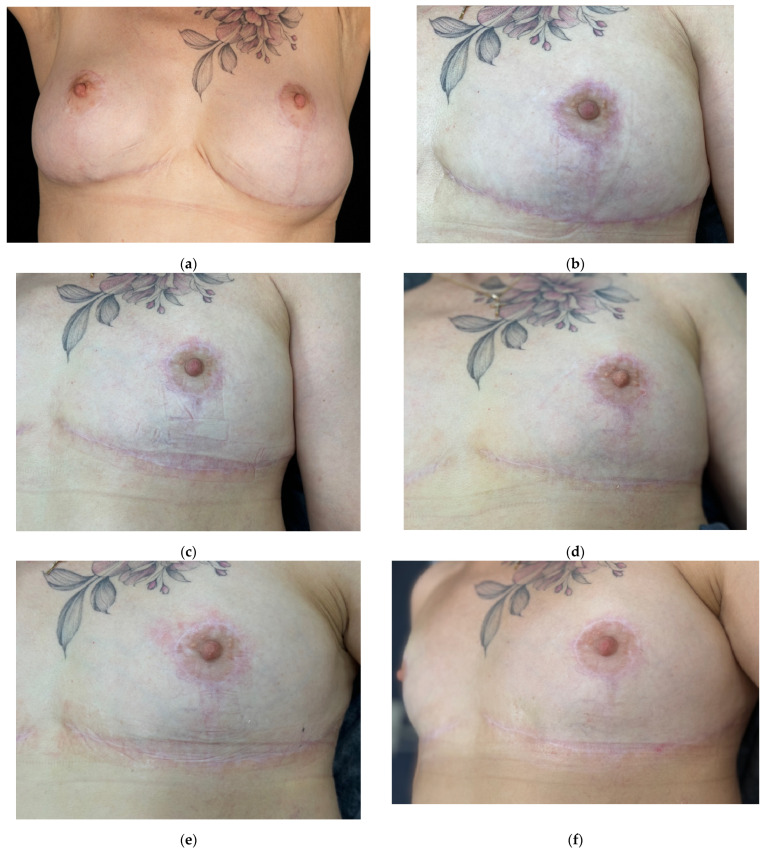

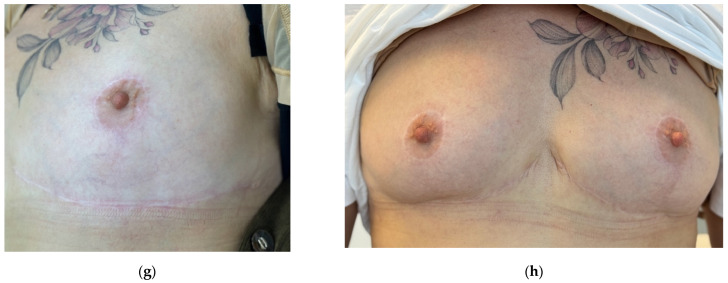
Clinical images documenting the prevention and treatment of hypertrophic scar formation after revision mammoplasty in a 56-year-old female patient. Time points: (**a**) baseline (pre-revision surgery); (**b**) 4.5 months after surgery (before the start of treatment); (**c**) 5.5 months after surgery (4 weeks after the first procedure); (**d**) 6.5 months after surgery (4 weeks after the second procedure); (**e**) 7.5 months after surgery (4 weeks after the third procedure); (**f**) 8 months after surgery (4 weeks after the fourth procedure); (**g**) 9 months after surgery (4 weeks after the fifth procedure); (**h**) 14 months after surgery (long-term outcome, five months after therapy).

A 56-year-old female patient presented with complaints of implant displacement and hypertrophic scarring after previous breast surgery (Figure 10a). She had undergone fat grafting 2.5 years earlier, followed by mastopexy and implant placement after developing lipogranulomas. 1 year after the operation, revision mammoplasty was performed due to the displacement of the implants and the formation of hypertrophic scars. The Vancouver scar score scale is 8.

In order to avoid previous complications, a third mammaplasty with implant removal was performed.

Standard postoperative care included topical corticosteroids, enzyme preparations, and later silicone sheets. At 4.5 months, the patient reported itching and tightness; the scar was erythematous with visible vessels (Figure 10b). The Vancouver scar score scale is 4.

To prevent hypertrophic scarring, 2 mL of 15% native collagen was injected intralesional. After the first session, the patient noted reduced symptoms and visible vascularity had decreased (Figure 10c). Two more sessions followed at four-week intervals using the same dosage. At the three-month follow-up, erythema and vascularity had significantly decreased (Figure 10e).

To consolidate results, two additional sessions with 2 mL of 7% collagen were performed at four-week intervals. By month five, the scar appeared thin, white, and normotrophic with no signs of progression (Figure 10g). The patient remained under clinical observation throughout the treatment period. At the 14-month follow-up, the scar remained flat and barely visible (Figure 10h). The Vancouver scar score scale is 1.

## 3. Discussion

Collagen plays a key role in modulating various regenerative mechanisms during cutaneous wound healing, including angiogenesis and re-epithelialization [29]. The application of type I collagen has been shown to accelerate healing, which in turn reduces the risk of hypertrophic scarring [4]. In Case 1, after using native collagen during all wound healing phases an aesthetically favorable scar formatted.

Timely progression through the wound healing phases is crucial. A delay in the transition between phases can disrupt scar maturation [30]. Angiogenesis peaks during the proliferative phase, typically within two weeks post-injury [31]. During this phase, granulation tissue is supplied with oxygen by newly formed vessels [32]. In granulation tissue, angiogenesis is determined by several factors, including the development of hypoxia gradients caused by local microvascular damage and cytokines. Hypoxia, caused by local microvascular damage, activates HIF-1α, a transcription factor that promotes angiogenesis. During proliferation, a disorganized vascular network is formed with highly convoluted vessels and numerous dead-end blood flow pathways. After that, the vascular network should mature, with the formation of larger vessels and regression of excess ones [33]. If hypoxia persists, pathological angiogenesis is sustained, further aggravating inflammation and promoting endothelial cell overproliferation—hallmarks of fibrosis [30].

The remodeling phase begins around week 3 and includes vascular regression, inflammation resolution, and replacement of granulation tissue with scar collagen [34,35]. Clinically, this phase corresponds to scar softening and fading [19]. An increase in redness or vascularity during this phase indicates a disruption in phase transition and increases the risk of pathological scarring, including the formation of hypertrophic scars.

Apoptosis of myofibroblasts in the remodeling phase is triggered by contact with type I collagen fibrils. When surrounded by fibrillar collagen, these cells lose their adhesive capacity and exit the cell cycle [30]. In fibrotic conditions, apoptosis is delayed, and fibroblasts continue to produce ECM proteins. This insight supports the clinical use of native collagen in managing hypertrophic scars.

In Cases 2–6, patients exhibited signs of disrupted wound healing, particularly persistent inflammation and vascularity during the remodeling phase. Even after 1 procedures of native collagen we can see improving wound healing.

Experimental studies have shown that electrospun nanofibrous scaffolds made of collagen and polyurethane reduce myofibroblast proliferation. Biomaterials may suppress fibroblast-to-myofibroblast conversion or decrease ECM protein secretion in hypertrophic scars [35,36].

In Case 4, hyperpigmentation was likely triggered by inflammation, supported by literature linking post-inflammatory hyperpigmentation to melanocyte stimulation via inflammatory mediators [37,38,39]. Type I collagen can bind inflammatory cytokines such as IL-1β, IL-6, and IL-8, promoting a healing microenvironment [40]. It also interacts with elastase and MMP-2, further regulating inflammation [41]. Treatment with 7% collagen reduced inflammation, normalized melanogenesis, and improved wound healing. In subsequent home care, along with a silicone-based cream, a cream with azelaic acid is recommended for a mild depigmenting effect, with an additional anti-inflammatory effect [42].

Collagen biodegrades at a rate proportional to tissue regeneration [43]. Thus, native collagen supports physiological ECM turnover, maintaining homeostasis and promoting optimal wound healing.

## 4. Conclusions

One of the most frequently reported complaints after blepharoplasty and other plastic surgeries is noticeable scarring [44,45]. Cosmetically unsightly scars may cause physical discomfort and psychosocial distress [46]. The final scar outcome depends on the wound healing process, which is influenced by wound healing process. Wound healing is a complex process that depends on the presence of various cell types, growth factors, cytokines, and ECM elements.

Tissue engineering advances have improved skin restoration via re-epithelialization, stromal-epidermal interactions, angiogenesis, and improved scar remodeling. Key cellular players include epidermal stem cells, fibroblast progenitors, adipose-derived stem cells, and bone marrow cells. These are regulated by growth factors such as EGF, FGF, PDGF, TGF, and ILs, underscoring the importance of ECM components and growth mediators in wound healing.

Regenerative medicine approaches are increasingly tailored to individual patients and have demonstrated high efficacy with minimal side effects [18]. Type I collagen serves as a natural substrate for cell adhesion and mediates multiple physiological interactions during healing, including angiogenesis and re-epithelialization [29]. Moreover, collagen binds and neutralizes excess MMPs, which are elevated in non-healing wounds. Various forms of collagen are being developed for use in skin and corneal wound healing [40]. Its role in guided tissue regeneration is well established [47].

Studies show that while collagen in normal skin is organized in a woven pattern, it is deposited in parallel fibers in scars. To counteract this, bioengineered collagen scaffolds aim to recreate the 3D architecture of native dermis and epidermis. These scaffolds provide a framework for neovascularization and possible implantation of keratinocytes in a more organized manner, as well as to ensure early wound coverage [48].

Acellular collagen-rich matrices degrade slowly and are replaced by newly synthesized ECM. They retain ligands for cell adhesion, aiding tissue integration [49].

Conventional scar treatments (silicone gel, pressure therapy, corticosteroids, surgical revision) often result in recurrence, side effects, or poor aesthetic outcomes [20]. Thus, more effective and safer regenerative solutions are being pursued. We have previously reported on the possibilities of using the product for skin rejuvenation in the management of patients with healing wounds [50].

In this case series, we demonstrated the value of native collagen for wound healing management.

The benefits of native collagen include:

- No atrophy or telangiectasia (common side effects of corticosteroids);
- Influence on both the wound and surrounding tissue for better healing;
- Physiological mechanism balancing ECM synthesis and degradation;
- Additional skin rejuvenation effects—valuable in aesthetic medicine;
- Physician supervision allows for early detection of abnormal scarring.

These features make native collagen a promising candidate for broader application in regenerative and aesthetic medicine.

## Figures and Tables

**Table 1:** Assessment of changes in the depth, width and redness of the scar using Antera 3D after trauma (clinical case 1).

Parameter	Day 17	Day 34	2.5 Months	3.5 Months	4.5 Months
Average width (mm)	2.06	1.73	1.62	1.6	0.792
Average depth (mm)	0.115	0.1	0.0981	0.097	0.0762
Redness					
Score	87	97	75	62	38
Minimum	18.7	24.4	27.8	24.4	22.2
Average	31.4	37.5	37.4	33.4	28.3
Maximum	53.6	58.1	57.8	53.4	41.2
Variation (%)	0.235	15.3	32.2	32.9	49.7

**Table 2:** Assessment of changes in the redness of the scar using Antera 3D after upper eyelid blepharoplasty (clinical case 2).

Parameter	4 Weeks	6 Weeks	9 Weeks	12 Weeks	16 Weeks
Score	93	114	112	89	84
Minimum	11.1	15.3	12.6	15.5	14.3
Average	29.2	34.3	34.4	30.7	29.1
Maximum	49.3	63.8	62.9	51.7	48.9
Variation (%)	0	0	0	0	0

**Table 3:** Assessment of changes in the volume and redness of the scar using Antera 3D after upper eyelid blepharoplasty (clinical case 3).

Parameter	5 Weeks	8 Weeks	11 Weeks	15 Weeks	19 Weeks
Volume (mm^3^)	12.1	11.9	11.5	11.4	10.1
Redness					
Score	121	106	105	93	87
Minimum	17.2	18	12.5	12.6	12.6
Average	38.4	32.9	32.4	31	29
Maximum	62	51.3	50	49.3	47.7
Variation (%)	0	0	0	0	0

**Table 4:** Assessment of changes in the depth, width and redness of the scar using Antera 3D after upper eyelid blepharoplasty (clinical case 4).

Parameter	6 Weeks	9 Weeks	12 Weeks	16 Weeks	20 Weeks
Pigmentation					
Score	169	142	134	131	129
Minimum	23.3	25.1	23.7	22.3	23.5
Average	42.2	41.2	38.5	38.7	40
Maximum	76.6	72.5	72.5	76.5	70.4
Variation (%)	0	0	0	0	0
Redness					
Score	111	110	114	109	103
Minimum	6.39	3.21	8.24	9.71	8.48
Average	25.3	27.5	30.9	28.4	28.4
Maximum	64.9	66.5	73.7	69.7	67.3
Variation (%)	0	0	0	0	0

## Data Availability

The data presented in this study are available upon request to the corresponding author due to confidentiality issues.

## Abbreviations

The following abbreviations are used in this manuscript:

Antera 3D	3D Skin Analysis Imaging System
Col α(I)	α chain of collagen I
ECM	Extracellular Matrix
EGF	Epidermal Growth Factor
FGF	Fibroblast Growth Factor
HIF-1α	Hypoxia-Inducible Factor 1-alpha
IL	Interleukin
MMP	Matrix Metalloproteinase
PDGF	Platelet-Derived Growth Factor
SPF	Sun Protection Factor
TGF-β	Transforming Growth Factor Beta
*VEGF*	Vascular Endothelial Growth Factor
VIM	Vimentin
